# Pullulan‐Collagen hydrogel wound dressing promotes dermal remodelling and wound healing compared to commercially available collagen dressings

**DOI:** 10.1111/wrr.13012

**Published:** 2022-04-18

**Authors:** Kellen Chen, Dharshan Sivaraj, Michael F. Davitt, Melissa C. Leeolou, Dominic Henn, Sydney R. Steele, Savana L. Huskins, Artem A. Trotsyuk, Hudson C. Kussie, Autumn H. Greco, Jagannath Padmanabhan, David P. Perrault, Alsu I. Zamaleeva, Michael T. Longaker, Geoffrey C. Gurtner

**Affiliations:** ^1^ Department of Surgery, Division of Plastic and Reconstructive Surgery Stanford University School of Medicine Stanford California USA; ^2^ TauTona Group Redwood City California USA

**Keywords:** collagen, extracellular matrix, hydrogels, inflammation, pullulan, wound healing

## Abstract

Biological scaffolds such as hydrogels provide an ideal, physio‐mimetic of native extracellular matrix (ECM) that can improve wound healing outcomes after cutaneous injury. While most studies have focused on the benefits of hydrogels in accelerating wound healing, there are minimal data directly comparing different hydrogel material compositions. In this study, we utilized a splinted excisional wound model that recapitulates human‐like wound healing in mice and treated wounds with three different collagen hydrogel dressings. We assessed the feasibility of applying each dressing and performed histologic and histopathologic analysis on the explanted scar tissues to assess variations in collagen architecture and alignment, as well as the tissue response. Our data indicate that the material properties of hydrogel dressings can significantly influence healing time, cellular response, and resulting architecture of healed scars. Specifically, our pullulan‐collagen hydrogel dressing accelerated wound closure and promoted healed tissue with less dense, more randomly aligned, and shorter collagen fibres. Further understanding of how hydrogel properties affect the healing and resulting scar architecture of wounds may lead to novel insights and further optimization of the material properties of wound dressings.

## 1 INTRODUCTION

Skin serves an essential role as a protective barrier against pathogens, water loss, as well as chemical and physical insults.^1^ Following cutaneous injury, wound healing progresses through a coordinated cascade of molecular and cellular processes to repair the damaged tissue.^2^ The fundamental objectives of wound healing therapies are twofold: to provide protection against external factors and to sustain optimal moisture levels within the wound bed.^3^, ^4^ Synthetic polymers such as poly‐(ethylene glycol) [PEG] have been used as wound dressings; however, they lack the biochemical properties for cellular interaction.^5^ Biologic scaffolds provide an ideal wound therapy that additionally provides a physiochemical mimetic of native ECM that further facilitates healing.^6^

Hydrogels are a biologic scaffold with a three‐dimensional structure that rapidly swells in water to form a semi‐solid. The water content of hydrogel matrices exceeds 90%, making them ideal for hydrating and maintaining a supportive (moist) environment within the wound bed.^7^ Over the past decade, there has been mounting evidence based on preclinical research findings that therapeutic hydrogels are highly effective at addressing concerns such as desiccation, bacterial infection, preventing debilitating scar formation, and promoting proper skin regeneration within the wound.^8^, ^9^ Furthermore, hydrogel dressings can be kept lyophilized, making them lightweight, portable, and shelf stable. They can be unpackaged in the clinic and rehydrated in saline at the point of care for use as a wound dressing.

The base materials used for biocompatible hydrogels studied to date include chitosan, hyaluronic acid, heparin, alginate, fibrin, and collagen.^5^ Collagen is possibly the most utilized base material for biologic scaffolds used in the clinic, as it is the primary organic constituent of native ECM, making it an attractive material for hydrogel synthesis.^10^ There are several commercially available collagen‐based hydrogels for wound healing in the current market, including Fibracol® Plus (90% collagen and 10% alginate) and Promogran™ (55% collagen, 45% oxidized regenerated cellulose [ORC]).^11^

Our group has engineered a novel pullulan‐collagen hydrogel with tunable, soft biomechanical properties and biocompatibility for cell‐based therapy encapsulation. We combined pullulan, a linear homopolysaccharide produced by the fungus *Aureobasidium pullulans* with Type 1 collagen, to develop a soft, biocompatible hydrogel that recapitulates the three‐dimensional organization of native ECM.^12^ This uniquely engineered collagen‐pullulan hydrogel confers several key advantages over traditional collagen hydrogels. First, pullulan‐collagen hydrogels have been shown to best approximate the porous ultrastructure of native reticular ECM based on comparison of fibre length and crosslinking distance. In a murine subcutaneous implantation model, the pullulan‐collagen hydrogel demonstrates retention of reticular architecture and cellular and cellular infiltration, indicating minimal rejection and an ideal biomaterial‐tissue integration.^13^ Moreover, altering the collagen: pullulan ratio enables fine tuning of the mechanical properties such as hydrogel stiffness and effective porosity with relative ease, allowing for small molecule and/or cell therapy delivery.^13^ This pullulan‐collagen hydrogel is now being manufactured according to Good Manufacturing Practices (GMP) by TauTona Group (Redwood City, CA), an ISO‐13485 certified facility, and referred to as the TauTona Wound Dressing (TWD).

**FIGURE 1:**
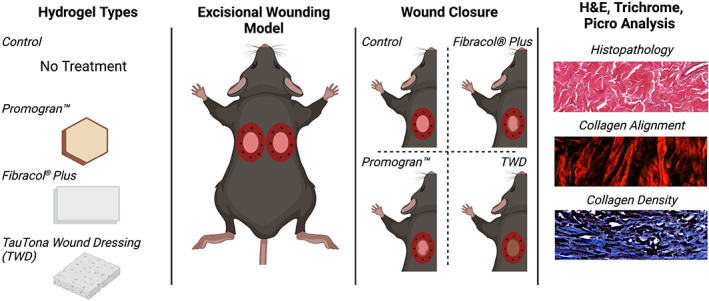
Overview schematic

In this study, we compared the efficacy of our pullulan‐collagen dressing to two commercially available predicate wound dressings Promogran™ and Fibracol® Plus, which both preclinical and clinical studies have previously shown to improve the likelihood of wound area reduction and wound resolution.^11^, ^14^ We utilized a mouse excisional wounding model and treated the wounds with either Promogran™, Fibracol® Plus, or our pullulan‐collagen dressing (TWD), as well as no treatment control wounds, and evaluated the wound response until closure. We also assessed the feasibility of applying each hydrogel dressing, including adherence, removal, and overall structural integrity. Finally, we performed histologic and histopathologic analyses on the explanted scar tissues to assess differences in collagen architecture and alignment, as well as immune cell infiltration (Figure 1).

## 2 METHODS

### 2.1 Animals

Eight‐ to twelve‐week‐old mice (eC57BL/6J; Jackson Laboratory, Bar Harbour, ME, www.jax.org) were housed in the Stanford University Veterinary Service Center and NIH and Stanford University animal care guidelines were followed. All procedures were approved by the university's Administrative Panel on Laboratory Animal Care (APLAC). The weight of each animal was recorded prior to operation (Day 0) and after completing the study (Day 14).

### 2.2 In vivo stented excisional wound model

Splinted full‐thickness excisional wounds were created as previously described by Galiano et al.^15^ Two full‐thickness dermal wounds of 6 mm diameter were created on the dorsum (left and right sides) of each mouse (C57BL/6J) using biopsy punches. A silicone ring was fixed to the dorsal skin around each wound using an adhesive glue (Vetbond, 3M, Saint Paul, MN). The ring was further reinforced using 8 interrupted 6‐0 nylon sutures placed around the outer edge of the ring to prevent wound contraction. Unlike humans, mice have a panniculus carnosus muscle underneath the dermis that contracts after injury. Thus, to mimic human‐like physiological wound healing that heals with granulation tissue and re‐epithelization, these silicone rings are used to stent the skin and prevent any contraction.

The wounds were either left untreated or treated with hydrogel dressings. Fibracol® Plus and Promogran™ dressings were trimmed to size with a 6 mm diameter biopsy punch to create a 6 mm patch. Following each product's respective ‘Instructions For Use’, patches were placed on top of the wound and then irrigated with sterile saline to saturate the hydrogel. For the TWD, in accordance with its ‘Instructions For Use’, the dressing was hydrated 5 min prior to application to saturate the hydrogel; the hydrogels were then cut to size with a 6 mm biopsy punch and then placed on the wounds. For all groups, the wounds were then covered with sterile secondary dressings. Digital photographs of the wounds were taken at the time of surgery and during every dressing change until the time of wound closure with a picture of a ruler next to the wound to standardize measurements. At the end of the study, the wounds were harvested on postoperative day 14 for histological evaluation.

### 2.3 Wound area analysis

For each wound, the wound edges were traced with the freehand tool in ImageJ and the area of the wound in pixels was analysed. As mentioned above, each ruler in the image was also measured to convert the number of pixels (pix length) corresponding to 1 mm. The pixel area was then multiplied by (1 mm/pix length)^2^ to convert the area into mm^2^. Each area was normalized to the corresponding initial wound area at postoperative day zero (POD 0).

### 2.4 Histologic analysis of collagen content and architecture

Explanted scar tissue and unwounded skin was harvested at the end of the study on day 14, fixed in 4% paraformaldehyde, dehydrated, and cryo‐embedded in optimal cutting temperature (OCT) compound for frozen sectioning on a microtome‐cryostat. Haematoxylin and Eosin (H&E) and Masson's Trichrome staining were performed according to the manufacturer's recommendations, and images were captured with a Leica Aperio AT2 digital whole slide scanner. We implemented an algorithm in MATLAB to automatically deconvolve the colour information of each Trichrome image.^16^ We determined a colour matrix based on the stain‐specific RGB light absorption of these samples:

$$C=\left[.80\;0.60\;0.10;\;.0.10\;0.70\;0.70;\;0.60\;0.32\;0.74\right]$$

The top two rows (semicolon denotes new row) correspond to stain‐specific RGB values of trichrome red and blue, respectively, and the columns represent the normalized vector values in the red, green, and blue channels. This algorithm allows for a robust and flexible method for objective immunohistochemical analysis of samples stained with up to three different colours.

Picrosirius Red (Sigma Aldrich) staining was also performed, and we utilized a Leica DM5000 B upright microscope for linear polarized light microscopy to capture images of the Picrosirius Red‐stained images. Polarized light was oriented to maximally display fibres parallel to the skin surface. Collagen fibre quantification was performed using CT‐FIRE and CurveAlign, an open‐source software package for automatic segmentation and quantification of individual collagen fibre (http://loci.wisc.edu/software/ctfire).^17^ Briefly, CurveAlign quantifies all fibre angles and the strength of alignment within an image, while CT‐FIRE analyses individual fibre metrics such as length, width, angle, and curvature. It also has the capability to extract other variables such as localized fibre density and the spatial relationship between fibre and the associated boundary. The average fibre parameters for each mouse were used for statistical analysis.

Finally, complexity and heterogeneity were measured using the ImageJ plug‐in FracLac.^18^ Briefly, FracLac analyses tissue morphology using fractional dimensions to determine the lacunarity (L) values using the subsample box counting scan (50 grid default sampling size, minimum pixel density threshold = 0, and rectangle subscan). L measures the amount of randomness or heterogeneity in a sample. A low L implies less heterogenous collagen fibre orientation.

### 2.5 Histopathology

All tissue sample histology slides were examined via light microscopy by a study pathologist. Wound sites were semi‐quantitively scored per criteria related to cell response, encompassing immune cell infiltration, as well as tissue response, encompassing neo‐vascularization, fibrosis, and fatty infiltration. Semi‐quantitative scores were assigned based on the representative site response observed over six noncontiguous representative high‐powered microscope fields at the wound surface. Wound sites were semi‐quantitatively scored per the criteria of Table 1. Semi‐quantitative scores were assigned based on the representative site response observed over 10 noncontiguous representative high‐powered microscope fields across the dermis. The groups were scored and documented by wound and animal in accordance with Table 1.

**TABLE 1:** Semiquantitative scoring criteria

Host response score	0 (none)	1 (minimal)	2 (mild)	3 (moderate)	4 (severe)
Cell response					
Polymorpho‐nuclear cells	0	1–5/hpf*	5–10/hpf	Heavy infiltrate	Packed
Lymphocytes	0	1–5/hpf	5–10/hpf	Heavy infiltrate	Packed
Plasma cells	0	1–5/hpf	5–10/hpf	Heavy infiltrate	Packed
Macrophages	0	1–5/hpf	5–10/hpf	Heavy infiltrate	Packed
Multinucleated giant cells	0	1–2/hpf	3–5/hpf	Heavy infiltrate	Sheets
Necrosis	0	Minimal	Mild	Moderate	Severe
Tissue response					
Neo‐vascularization	0	Minimal capillary proliferation, focal, 1–3 buds	Groups of 4–7 capillaries with supporting fibroblastic structures	Broad band capillaries with supporting fibroblastic structures	Extensive band of capillaries with supporting fibroblastic structures
Fibrosis	0	Narrow band	Moderately thick band	Thick band	Extensive band
Fatty infiltration	0	Minimal amount of fat associated with fibrosis	Several layers of fat and fibrosis	Elongated and broad accumulation of fat cells about the implant site	Extensive fat completely surrounding the implant

* hpf, high‐powered field (40× objective).

### 2.6 Assessing dressing performance

Dressing performance was assessed by comparing the adherence, removal, and integrity of each dressing during each dressing change. Adherence was defined as the ability of the dressing to stay on the wound after dressing removal. High adherence indicated that the hydrogel could not be easily removed from the wound. Non to low adherence indicated that the wound dressing was either stuck to the secondary dressing or did not adhere to the wound, allowing easy removal of the hydrogel. After assessing adherence, each dressing was then carefully and fully removed from the wound with gentle saline irrigation and gentle peeling with forceps. Each parameter was represented as a percentage of total observations through the study within one group of interest.

### 2.7 Immunofluorescent staining

Immunofluorescent staining was performed using a primary antibody targeting F4/80 (1:100 dilution; Abcam, ab6640). The percentage of fluorescent area was quantified using a custom MATLAB image processing code written by the authors and previously published.^19^ All immunofluorescent images shown are representative images.

### 2.8 Statistical analysis

Statistical analysis was performed in Prism8 (GraphPad, San Diego, California) using either a two‐way or one‐way analysis of variance (ANOVA) with Tukey's multiple comparisons test. Data are presented as means ± SEM. Values of *p < 0.05 were considered statistically significant.

### 2.9 Electronic notebook

No electronic notebook was used.

## 3 RESULTS

### 3.1 Pullulan‐collagen hydrogel dressing accelerates healing of murine wounds compared to predicate devices

**FIGURE 2:**
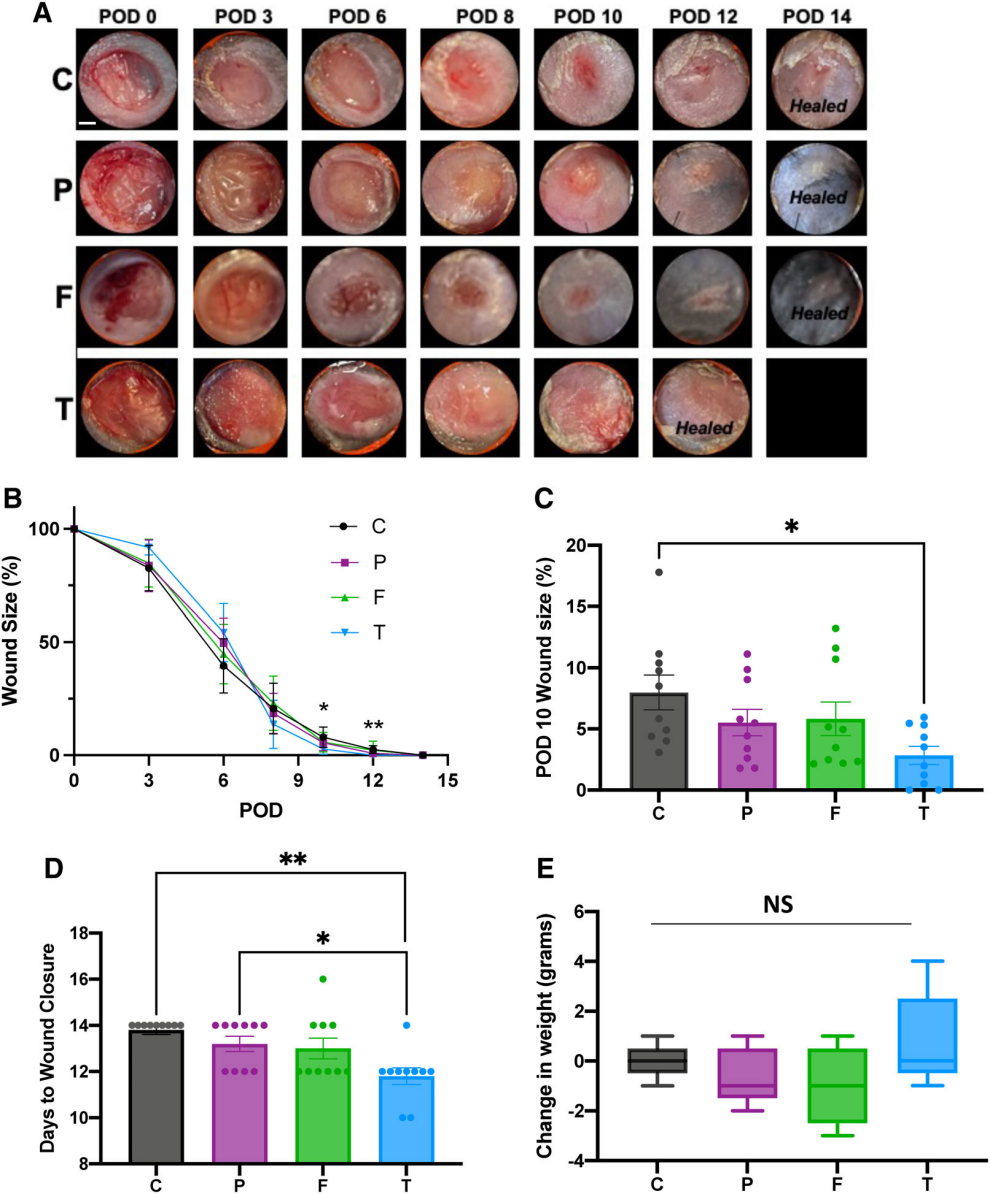
(A) Representative images of the wound are over time by treatment group, where C = Control; P = Promogran™; F = Fibracol® Plus; T = TauTona Wound Dressing. POD, postoperative day. *Healed* = healed wound that has closed. (B) Quantification of wound area over time by treatment group. (C) Wound area size at POD10 (*n* = 10/group). (D) Days until complete wound closure by treatment group (*n* = 10/group). (E) Change in mouse weight from pre‐op (Day 0) to study completion (Day 14) (*n* = 5/group). Scale bar: 1 mm. Statistical analysis was performed using analysis of variance (ANOVA) with Tukey's multiple comparisons test

To measure the effect of each hydrogel treatment on wound healing, we assessed wound area change over time by analysing digital photographs that were taken during each dressing change. Representative images of the wounds over time are shown in Figure 2A. The initial wounds at postoperative day (POD) 0 are observed. Each wound remains moist until granulation tissue built up around PODs 8–10. After granulation tissue formed, the wounds were no longer moist. As re‐epithelialization occured, the wounds then became closed and fully healed. The quantification of the wound area changes over time is shown in Figure 2B. The wound size is represented as the average size of 10 wounds per treatment group (5 mice per group, 2 wounds per mouse). At PODs 10 and 12, the wound areas were significantly smaller in wounds treated with the pullulan‐collagen TWD compared to Control wounds (Figure 2B). Both Promogran™ and Fibracol® Plus treatment groups demonstrated wound sizes that were not significantly different from either the TWD or Control wounds across all time points. Since the absolute wound size percentages were difficult to fully appreciate in Figure 2B, the wound percentage sizes at PODs 10 and 12 are shown as individual bar graphs (Figure 2C, Supplementary Figure S1), showing that the TWD‐treated wounds had significantly smaller wound sizes compared to Control group at both POD10 (*p < 0.05) and POD12 (**p < 0.01). Furthermore, Fibracol‐treated wounds were also significantly smaller than Control wounds at POD12 (**p < 0.01). At both POD10 and POD12, the TWD‐treated wounds were also not significantly different from either the Promogran™ and Fibracol® Plus Groups, which were also not statistically significant from the Control group. These results show that wound closure dynamics are relatively similar for all treatment groups, but that the TWD increases the wound closure rate at later stages of the healing process when compared to the Control and Promogran™ groups.

We then assessed the digital photographs of each mouse wound to determine the average number of days before complete wound closure for each treatment group (Figure 2D). The days to closure of the wounds treated with the TWD (average ~11.2 days) was significantly shorter than the days to wound closure of wounds treated with both Promogran™ (~13.2 days; *p < 0.05) and Control wounds (~13.8 days; **p < 0.01; *n* = 10 per group). The days to closure of the wounds treated with Fibracol® Plus was approximately 13 days, which was not significantly different from any of the other groups. Our data indicate that the TWD promotes days to wound closure at a similar level as Fibracol® Plus dressing, but faster than Control and Promogran™. All three dressings maintained an optimal moist microenvironment at the wound surface that was conducive to granulation tissue formation and re‐epithelialization to enable healing to proceed at a rapid rate.

The weight of each animal was recorded prior to operation (day 0) and after completing the study (day 14). On average, mice weighed about 16–17 grams on days 0 and 14, which indicated a healthy mouse weight (Figure 2E). As compared to the Control group (C), topical treatment of the wounds using Promogran™, Fibracol® Plus, or TWD did not result in any adverse or treatment‐related body weight effects. There was no difference in weight change between the treatment groups, indicating that none of the treatment groups caused any negative effects on animal health.

### 3.2 Pullulan‐collagen hydrogel dressing improves collagen architecture in murine wounds

**FIGURE 3:**
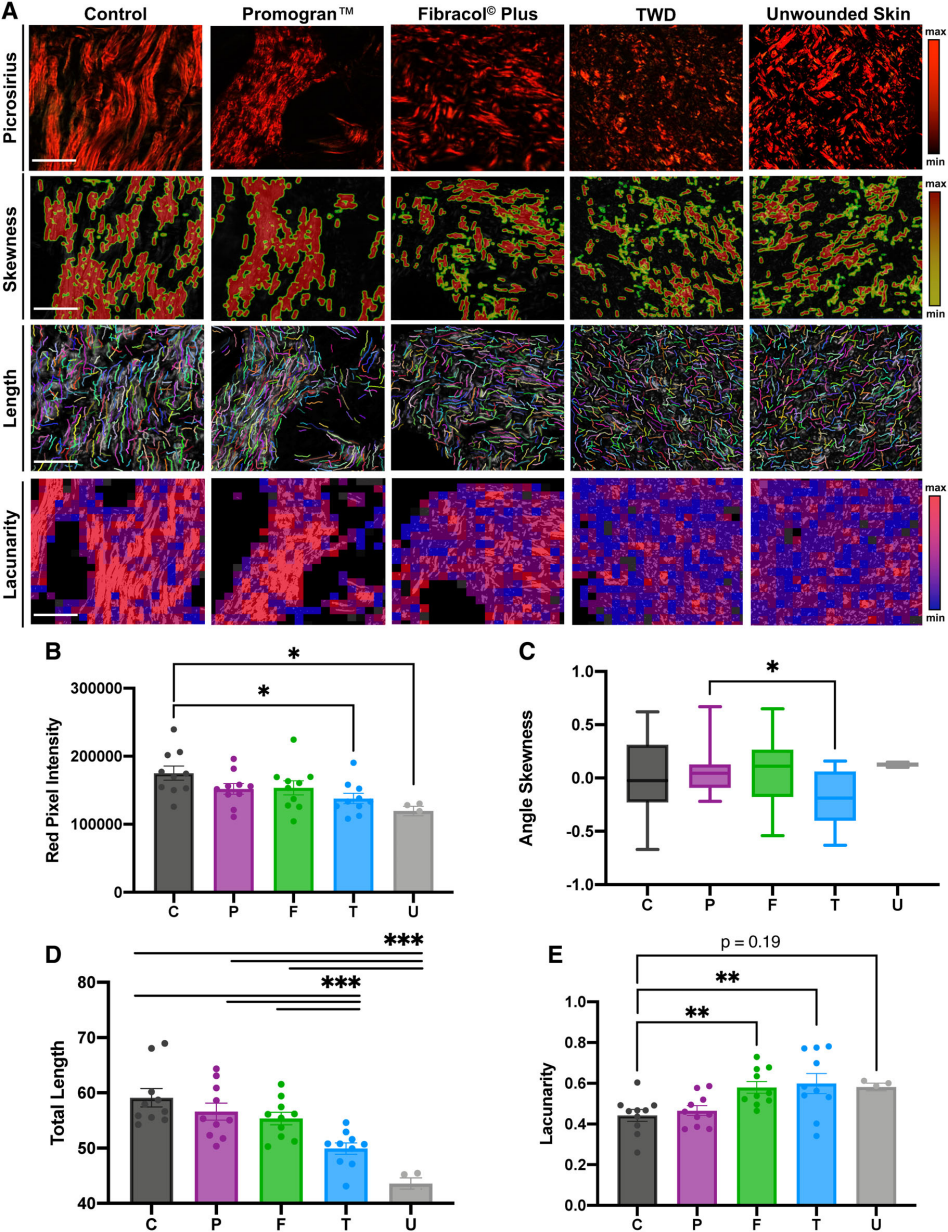
(A) Picrosirius red staining and comparison of Control, Promogran™, Fibracol Plus, TWD‐treated wounds, and unwounded skin, using collagen algorithms CurveAlign, CT‐Fire, and FracLac. Scale bars: 200 μm. Colour bars indicate colours corresponding to minimum and maximum intensities. Quantification of (B) collagen fibre pixel intensity, (C) fibre angle skewness, (D) fibre length, and (E) tissue lacunarity. *n* = 10 for Control, Promogran™, Fibracol Plus, TWD groups; *n* = 4 for unwounded skin group. Statistical analysis was performed using analysis of variance (ANOVA) with Tukey's multiple comparisons test

We next evaluated the collagen architecture of healed wounds in the Control, Promogran™, Fibracol® Plus, or TWD groups at day 14 (Figure 3A). We also evaluated the collagen architecture of unwounded murine skin as an additional control. A detailed quantitative assessment of the collagen architecture of the wounds was analysed using the software algorithms CT‐Fire, CurveAlign, and FracLac, which have been previously developed for analysis of collagen fibre properties in histologic images.^20^, ^21^, ^22^ Utilizing this array of metrics, the fibre length, angle skewness, red pixel intensity, and fibre lacunarity were analysed. We used picrosirius red staining to evaluate the collagen density and orientation of the scars in each group. Both TWD‐treated wounds and unwounded skin demonstrated significantly diminished red pixel intensity compared to untreated control wounds (*p < 0.05), indicating a lower amount of mature collagen within the healed TWD‐treated scars with similar intensity to unwounded skin (Figure 3B). We used the CT‐FIRE algorithm to perform single fibre extraction and analysis in the histologic images as well as CurveAlign, a curvelet transform‐based fibrillar collagen quantification platform to analyse fibre alignment as a surrogate parameter for fibrosis. TWD‐treated wounds showed significantly more random alignment compared to Promogran™‐treated wounds (*p < 0.05; Figure 3C). Further, CT‐FIRE showed that both TWD‐ treated wounds and unwounded skin demonstrated significantly shorter fibre lengths compared to Fibracol® Plus, Promogran™, and Control wounds (*p < 0.05) (Figure 3D). Finally, we used FracLac analysis to assess the complexity and heterogeneity of the healed wounds in all groups. TWD and Fibracol® Plus‐treated wounds displayed significantly greater lacunarity compared to control wounds (**p < 0.01), indicating a more heterogeneous collagen fibre network orientation (Figure 3E). Lacunarity measures the number of gaps in the tissue and is thus a marker of tissue density. We found that TWD‐treated wounds possessed greater porosity, more similar to a dermal‐like architecture.

Since fibrotic tissue is typically characterized by long, densely aligned fibres with high intensity, we observed that TWD significantly promoted restoration of more unwounded skin architecture, characterized by shorter, more randomly aligned fibres with lower intensity of collagen signal. Across these metrics, TWD improved healing compared to control wounds. For the fibre length metric specifically, TWD significantly decreased fibre length to levels more similar to unwounded skin compared to both Fibracol® Plus and Promogran™ groups. Among all comparisons (i.e., red pixel intensity, angle skewness, fibre length, and lacunarity), TWD displayed the most similarities to the unwounded murine skin group. Thus, these unbiased computer algorithms quantified that treatment with TWD promoted even more positive benefits to the collagen structure of healed skin after wounding compared to both control wounds and wounds treated with predicate devices. Taken together, these results suggest that TWD promoted shorter and more randomly aligned collagen in the wound bed, more like the typical basket weave‐like collagen fibre networks resembling the physiologic dermal collagen architecture of unwounded murine skin.

**FIGURE 4:**
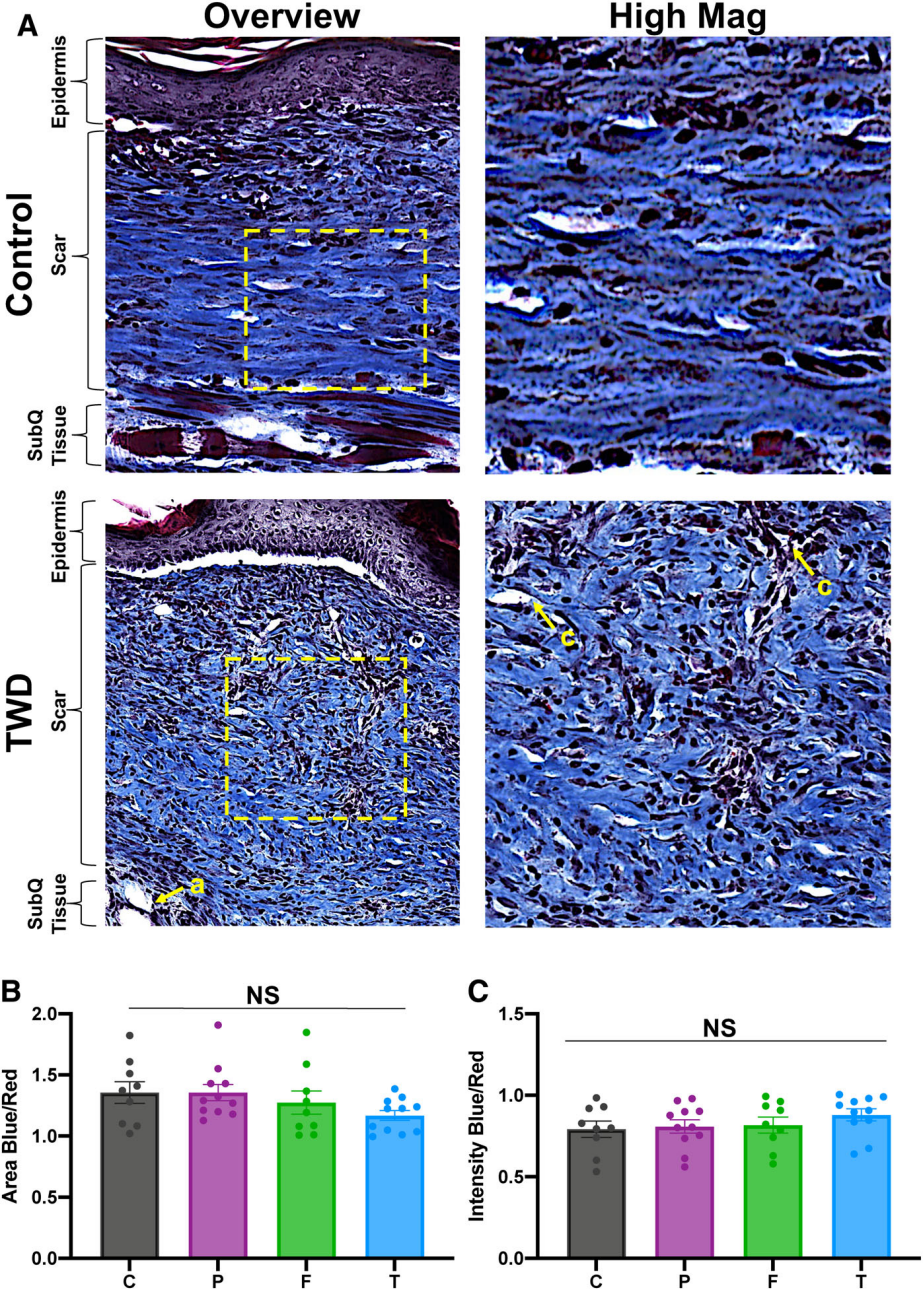
(A) Masson's trichrome staining of representative tissue sections showing dermal structure of control and TWD‐treated wounds. (B) Analysis for total area positive for collagen (area blue) and (C) total proportion of mature collagen (blue intensity). Scale Bar: 300 μm. *n* = 10 for all groups. Statistical analysis was performed using analysis of variance (ANOVA) with Tukey's multiple comparisons test. Yellow arrows denote structures such as adipose tissue (a) and capillaries (c)

Dermal structure of murine scar tissue was also analysed using histological (Trichrome) staining (Figure 4A). Trichrome staining provided additional support to the picrosirius red staining analysis results, showing a more randomly aligned, basket weave‐like collagen fibre network in the TWD‐treated scars, with vascularization present throughout the matrix. In contrast, control scar wounds were characterized by the presence of large, long bundles of avascular collagen. The collagen area and proportion of mature collagen were similar among all groups, with TWD‐treated wounds trending towards decreased total area positive for collagen (Figure 4B, C). We also found that the total scar area was similar between all groups (Figure S2).

### 3.3 Pullulan‐collagen hydrogel dressing demonstrates clinical feasibility and ease of use

We assessed the feasibility of applying each dressing in a clinical setting by comparing the adherence, removal, and integrity of each hydrogel dressing during each dressing change (Table 2). Promogran™ and Fibracol® Plus had a higher percentage (~63% and ~89%, respectively) of displaying non to low adherence, largely because they remained adherent to the secondary dressings during removal. If not adhered to the dressing, Promogran™ was also then more likely to break apart into small pieces that were tightly adhered to the wound and were thus difficult (not easy) to remove. In contrast, the TWD had a higher average percentage (~94%) of non to low adherence, meaning that the TWD was easier to remove from the wound after removing the secondary dressing. In the cases that the TWD did not adhere to the wound, it was also generally not adherent to the secondary dressing. Instead, we found that the TWD could easily be removed from the wound due to its relative thickness and increased hydration.

**TABLE 2:** Wound dressing adherence, ease of removal, and integrity

Treatment group	Adherence (%)		Removal (%)		Integrity (%)	
	Non to low	High	Easy	Not easy	Intact	Damaged
Promogran™	63	37	44	56	4	96
Fibracol® Plus	89	11	76	24	52	48
TWD	94	6	92	8	98	2

The TWD duration specified per design requirements was defined as the TWD's ability to cover the wound for up to 72 h while maintaining its initial integrity. To validate this specification, the dressings were changed every third day (72 h after dressing application) for 6 days. After day 6, the dressings were changed every other day to ensure that the wounds remained well splinted with silicone rings (the splints tend to need re‐suturing after the first 6 days). The integrity of the TWD was mostly intact (98%), demonstrating that the TWD can cover the wound up to 72 h while maintaining its initial integrity. The Fibracol® Plus dressing, on average, exhibited physical properties in between that of the Promogran™ and TWD. For example, about half of the hydrogels were damaged during the dressing changes, while the other half were intact. Additionally, the Fibracol® Plus dressing removal was relatively easy (76%), which was a higher percentage than in Promogran™ (44%) but lower than in TWD (92%; Table 2).

Overall, the TWD demonstrated the strongest structural integrity during dressing changes and was the easiest to remove. The Promogran™ dressing was usually destroyed or highly adherent either to the dressing or to the wound, and the Fibracol® Plus performed superior to Promogran™ but not as well as TWD. These results support the clinical feasibility of TWD and its relative ease of use compared to other hydrogel dressings currently on the market.

## 4 HISTOPATHOLOGY EVALUATION

For histopathology analysis, tissue from each of the 10 wounds from either control, Promogran™, Fibracol® Plus, and TWD groups were explanted and subjected to Haematoxylin and Eosin (H&E) staining. These were then imaged with high magnification and subjected to histopathological analysis to interrogate the types of cells and tissue response within each group.

**FIGURE 5:**
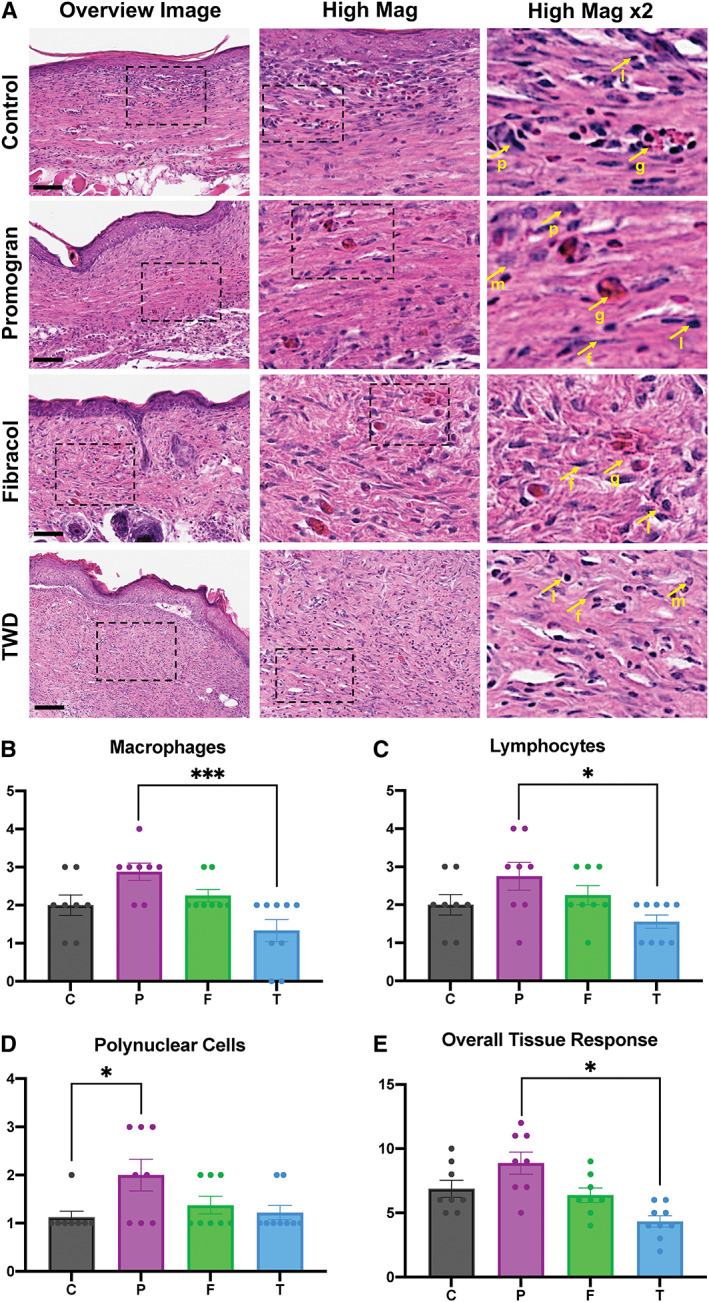
(A) Representative H&E images of healed murine excisional wounds showing cells (nuclei in purple) and extracellular matrix (pink) in all groups. Box indicates area chosen for higher magnification images. Arrows indicate; p = polymorphonuclear cells; m = macrophages; l = lymphocytes; f = fibroblasts (indicative of fibrosis); g = giant cells. C = Control Wounds; P = Promogran™‐treated wounds; F = Fibracol® Plus; T = TauTona Wound Dressing. Scale bars: 150 μm. Each image was semi‐quantitatively analysed with histopathology to determine the presence of (B) macrophages, (C) lymphocytes, (D) polynuclear cells, and (E) overall tissue response. *n* = 10 for all groups. Statistical analysis was performed using analysis of variance (ANOVA) with Tukey's multiple comparisons test

Wound sites were semi‐quantitatively scored as described in the Methods section. The results are presented in Figure 5. In the Promogran™ wound groups, medium to severe presence of polymorphonuclear cells, lymphocytes, macrophages, giant cells, neovascularization, and fibrosis was observed, with no presence of plasma cells, necrosis, or fatty infiltration observed. These could be seen in the representative histological images shown in Figure 5A. In both control and Fibracol® Plus‐treated wounds, mild to moderate polymorphonuclear cells, lymphocytes, macrophages, giant cells, neovascularization, and fibrosis were observed, with no presence of plasma cells, necrosis, or fatty infiltration observed. In TWD‐treated wounds, mild to minimal polymorphonuclear cells, lymphocytes, macrophages, giant cells, neovascularization, and fibrosis were observed, with no presence of plasma cells, necrosis, or fatty infiltration observed.

Specifically, TWD‐treated wounds demonstrated a significantly decreased number of macrophages (Figure 5B), lymphocytes (Figure 5C), and overall tissue response (Figure 5E) compared to Promogran™‐treated wounds. In addition, Promogran™‐treated wounds demonstrated an increased number of polynuclear cells compared to control wounds (Figure 5D). Overall, a total reactivity grade was calculated as 0 for both TWD and Fibracol® Plus compared to Control Wounds, indicating minimal or no reaction. In contrast, a total reactivity grade was calculated as 4.75 for Promogran™ compared to Control Wounds, indicating a mild reaction. We confirmed these results using immunohistochemical staining of F4/80 macrophages, a unique marker for cells of the mononuclear phagocyte lineage in mice, and found a significant reduction in relative F4/80 expression in the TWD group compared with the Promogran™‐treated wounds (Figure S3).

## 5 DISCUSSION

Many studies have investigated the role of various biologic hydrogel scaffolds to promote wound healing, including from our group, which has engineered a pullulan‐collagen hydrogel with tunable mechanical properties and enhanced biocompatibility for wound repair and regeneration.^4^, ^12^, ^13^, ^23^, ^24^, ^25^ However, previous studies have not directly compared the efficacy, feasibility, and resulting scar histology among commonly used hydrogel wound dressings using advanced collagen architecture algorithms. Here, we found that continuous treatment of murine excisional wounds with the TWD for 14 days did not result in any adverse reactions within the healed wound tissue. TWD accelerated wound closure statistically faster than both Promogran™ and Control groups. Utilizing unbiased collagen analysis, TWD was shown to promote tissue with less dense, more randomly aligned, and shorter collagen fibres with lower collagen intensity, more similar to the natural ‘basket‐weave’ architecture of unwounded skin. Histopathologic analysis showed preservation of dermal anatomical structures and minimal to no reaction of the TWD compared to predicate devices (Promogran™ and Fibracol® Plus) as well as Control wounds.

Promogran™ and Fibracol® Plus have both been investigated extensively in preclinical and clinical studies. In a randomized study, Promogran™ was shown to improve wound closure in patients with diabetic foot ulcers and reduce incidence of infections by 31% compared to standard of care.^26^ Further, another study investigating the influence of Promogran™ on diabetic foot ulcers showed that patients had significantly less gelatinase, elastase, and plasmin in the wound as well as reductions in matrix metalloproteinase‐2 compared to those treated with hydrocolloid dressings.^27^ Fibracol® Plus has been shown in preclinical studies to display a matrix structure analogous to intact, native, dermal collagen. In addition, it has been shown to significantly promote fibroblast proliferation in comparison to ORC/collagen matrices.^28^ A clinical study of 75 patients with diabetic foot ulcers saw an 18% improvement in wound area reduction in patients treated with Fibracol® Plus compared to controls. Complete healing was achieved in 48% of the collagen‐alginate dressing group and 36% of the control dressing group.^14^ Based on the results of this study, we believe the TWD could provide equal, if not improved, benefits over already established collagen hydrogels that have shown these strong clinical results.

Our group has previously shown that collagen‐pullulan scaffolds recapitulate a porous dermal‐like architecture and significantly augment normal cutaneous wound repair. Here, our findings build upon our previous in vitro and in vivo analyses demonstrating the biocompatibility of these pullulan‐collagen hydrogels.^12^ These hydrogels also provide an additional benefit to conventional hydrogel scaffolds in that they can support the growth of multiple cell types including fibroblasts, endothelial cells, bone marrow‐derived mesenchymal stromal cells (MSCs), and adipose‐derived stem cells (ASCs) with negligible cytotoxicity.^12^, ^13^, ^23^, ^25^, ^29^ A subset of ASCs (DPP4^+^/CD55^+^) with enhanced regenerative potential were successfully seeded in our biomimetic pullulan‐collagen hydrogel and tested in a diabetic murine excisional wound healing model. The ASC seeded pullulan‐collagen treatment demonstrated enhanced time to closure and improved dermal recover compared to control.^29^ We further validated our pullulan‐collagen hydrogel scaffold in its ability to delivery bone marrow‐derived MSCs in a murine excisional wound healing model, which demonstrated that wounds treated with MSC‐seeded hydrogels showed significantly accelerated healing and a return of skin appendages.^25^ Finally, we showed that adipose‐derived stromal cells seeded in the pullulan‐collagen hydrogel improved healing in a murine burn model. Burn wounds treated with ASC‐seeded pullulan‐collagen hydrogels significantly reduced wound closure time, reduced scarring, and reconstructed collagen networks.^23^ These hydrogels can also be incorporated with a small molecule inhibitor for pharmacologic improvement of healing.^20^, ^30^ Thus, while our biomimetic pullulan‐collagen hydrogel improves healing alone, it also has the added potential of providing a functional niche capable of harbouring therapeutic cells or serving as a drug carrier. This can result in additionally augmented wound healing, further supporting the beneficial and additive capabilities of pullulan‐collagen hydrogel scaffolds for wound healing applications.

In our study, we also found that that TWD‐treated wounds had diminished macrophage infiltration and overall tissue response within the healed scar. Macrophages have been to play an important role in inflammation, healing, and hypertrophic scar formation.^31^, ^32^ A previous study by Wong et al. showed that pullulan‐collagen hydrogel dressings attenuated macrophage (F4/80^+^) recruitment in hydrogel‐treated wounds compared with Control wounds.^12^ Further, Cheng et al. observed fewer macrophages in the wound bed of pullulan‐gelatin hydrogel scaffold‐treated wounds compared to controls.^33^ Both studies showed improved healing with their respective pullulan hydrogels, indicating that reducing the number of infiltrating macrophages and inflammatory cells within the healing wound bed leads to improved healing outcomes, including mitigation of scar formation. Although macrophages are essential in wound repair, it has been hypothesized that heavy recruitment of these cell types may delay matrix deposition during the early phases of wound healing due to their phagocytic and enzymatic activity.^34^ ^,^^35^ The pullulan‐collagen hydrogel reduced macrophage infiltration and overall tissue response, which led to accelerated wound closure and improved tissue repair. Future studies will need to be performed to further interrogate the mechanisms driving the potential anti‐inflammatory properties of pullulan‐collagen hydrogels.

## CONFLICT OF INTEREST

Kellen Chen, Michael T. Longaker, and Geoffrey C. Gurtner serve as consultants for the TauTona Group. All other authors declare no competing interests.

## AUTHOR CONTRIBUTION

Kellen Chen, Dharshan Sivaraj, Alsu I. Zamaleeva, and Geoffrey C. Gurtner designed the study. Kellen Chen, Dharshan Sivaraj, Michael F. Davitt, Dominic Henn, Melissa C. Leeolou, Sydney R. Steele, Savana L. Huskins, Artem A. Trotsyuk, Hudson C. Kussie, Autumn H. Greco, David P. Perrault, and Jagannath Padmanabhan performed the animal experiments and data analysis. Kellen Chen, Dharshan Sivaraj, and Melissa C. Leeolou wrote the manuscript. Geoffrey C. Gurtner helped revise and edit the manuscript.

## Supporting information


**Figure S1**Wound area size at POD12. *n* = 10 for all groups. Statistical analysis was performed using analysis of variance (ANOVA) with Tukey's multiple comparisons test.
**Figure S2**. Total scar area measurement at POD14. *n* = 10 for all groups. Statistical analysis was performed using analysis of variance (ANOVA) with Tukey's multiple comparisons test.
**Figure S3**. F4/80 immunofluorescent staining of cross‐sectional murine excisional wounds. Relative staining intensity shows a reduction in F4/80 in the TWD group (20.9 ± 8.6) as compared with the Promogran™ group (96.7 ± 27.6)‐treated wounds. *p = 0.0387. *n* = 5 for all immunofluorescent groups. Data are means ± one SEM. Statistical analysis was performed using analysis of variance (ANOVA) with Tukey's multiple comparisons test.

## Data Availability

Data available on request from the authors
