# Supplementary figures and images for: Pullulan‐Collagen hydrogel wound dressing promotes dermal remodelling and wound healing compared to commercially available collagen dressings
Source: Wound Repair Regen. 2022 Apr 18;30(3):397–408. doi: 10.1111/wrr.13012 (PMC9321852; doi:10.1111/wrr.13012)

# Supplementary Figure 1

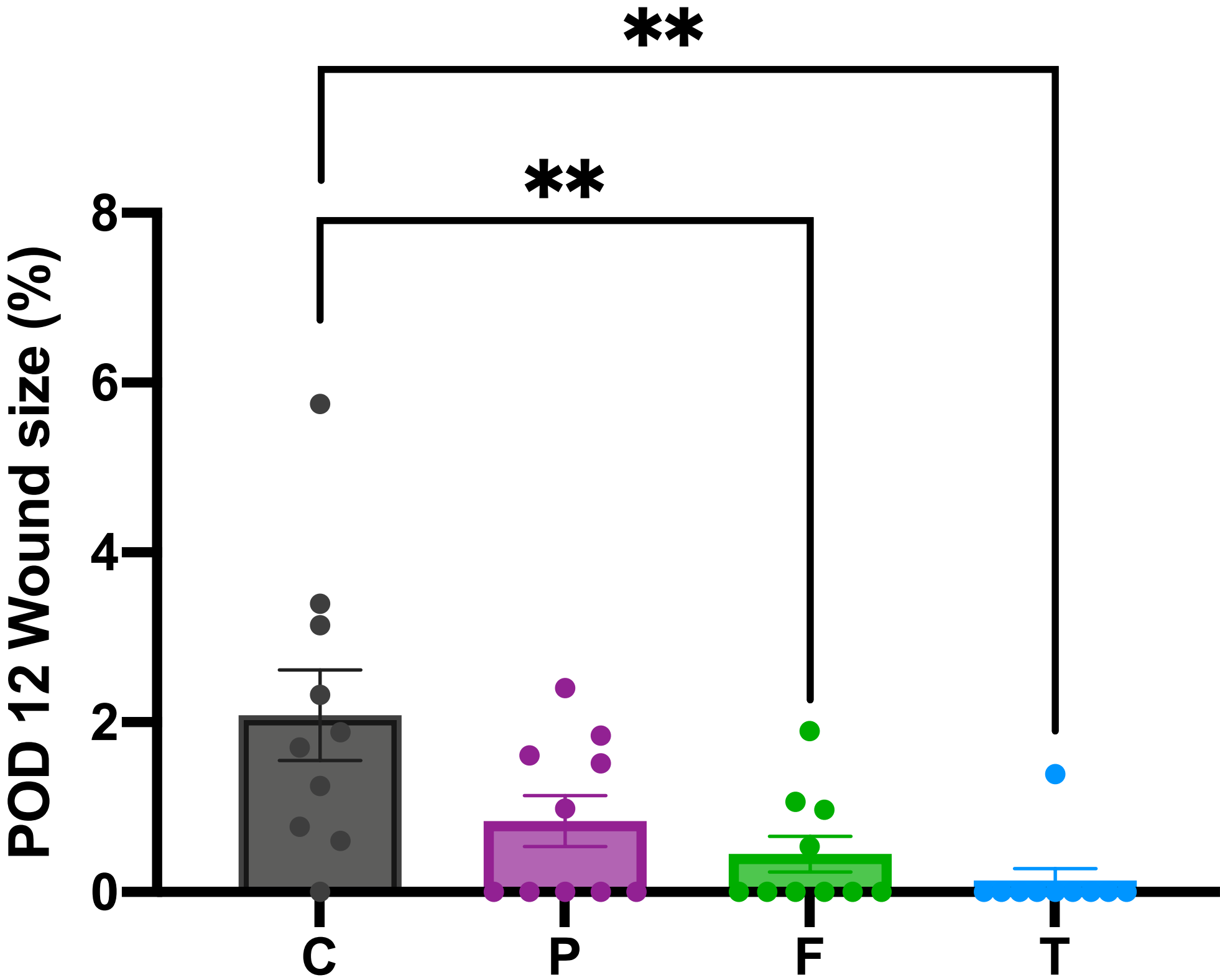

# Supplementary Figure 2

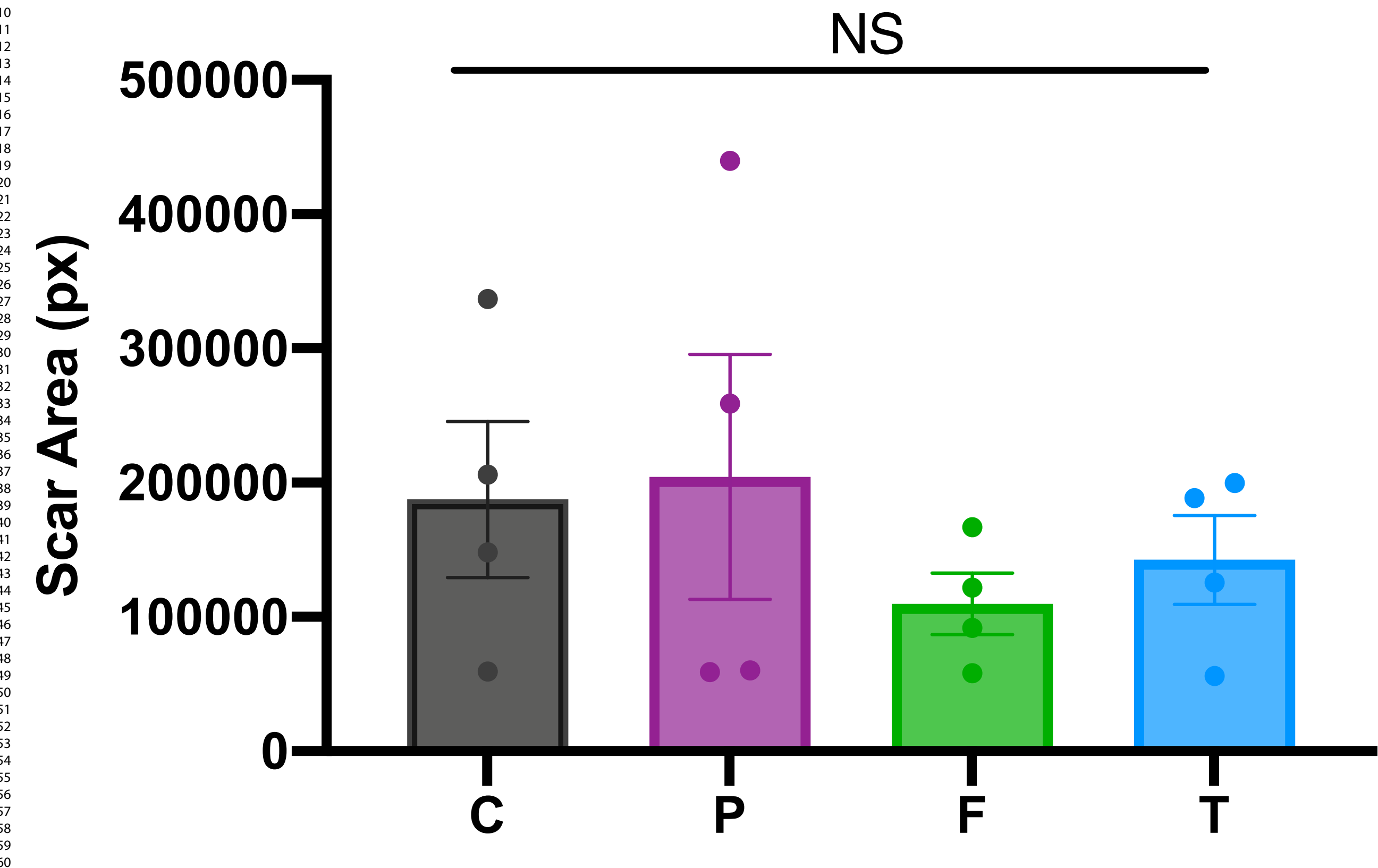

# Supplementary Figure 3

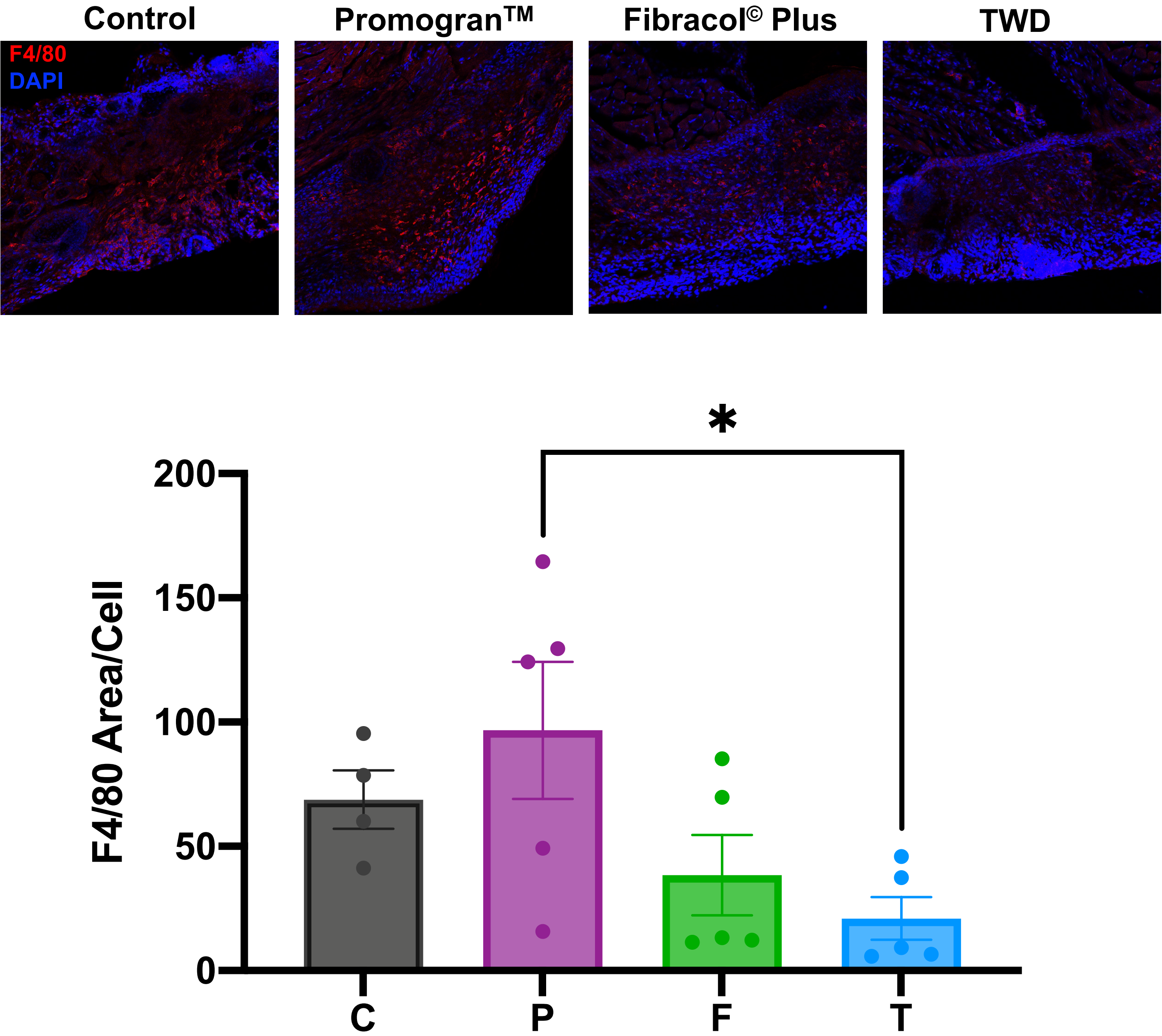

Supplement: Supplementary file 1 — Figure S1Wound area size at POD12. n = 10 for all groups. Statistical analysis was performed using analysis of variance (ANOVA) with Tukey's multiple comparisons test. Figure S2. Total scar area measurement at POD14. n = 10 for all groups. Statistical analysis was performed using analysis of variance (ANOVA) with Tukey's multiple comparisons test. Figure S3. F4/80 immunofluorescent staining of cross‐sectional murine excisional wounds. Relative staining intensity shows a reduction in F4/80 in the TWD group (20.9 ± 8.6) as compared with the Promogran™ group (96.7 ± 27.6)‐treated wounds. *p = 0.0387. n = 5 for all immunofluorescent groups. Data are means ± one SEM. Statistical analysis was performed using analysis of variance (ANOVA) with Tukey's multiple comparisons test. [file WRR-30-397-s001.pdf]
